# Mapping Soil Transmitted Helminths and Schistosomiasis under Uncertainty: A Systematic Review and Critical Appraisal of Evidence

**DOI:** 10.1371/journal.pntd.0005208

**Published:** 2016-12-22

**Authors:** Andrea L. Araujo Navas, Nicholas A. S. Hamm, Ricardo J. Soares Magalhães, Alfred Stein

**Affiliations:** 1 Faculty of Geo-information Science and Earth Observation (ITC), University of Twente, AE, Enschede, The Netherlands; 2 UQ Spatial Epidemiology Laboratory, School of Veterinary Science, The University of Queensland, Gatton QLD, Australia; 3 Child Health and Environment Program, Child Health Research Centre, The University of Queensland, South Brisbane QLD, Australia; Common Heritage Foundation, NIGERIA

## Abstract

**Background:**

Spatial modelling of STH and schistosomiasis epidemiology is now commonplace. Spatial epidemiological studies help inform decisions regarding the number of people at risk as well as the geographic areas that need to be targeted with mass drug administration; however, limited attention has been given to propagated uncertainties, their interpretation, and consequences for the mapped values. Using currently published literature on the spatial epidemiology of helminth infections we identified: (1) the main uncertainty sources, their definition and quantification and (2) how uncertainty is informative for STH programme managers and scientists working in this domain.

**Methodology/Principal Findings:**

We performed a systematic literature search using the Preferred Reporting Items for Systematic reviews and Meta-Analysis (PRISMA) protocol. We searched Web of Knowledge and PubMed using a combination of uncertainty, geographic and disease terms. A total of 73 papers fulfilled the inclusion criteria for the systematic review. Only 9% of the studies did not address any element of uncertainty, while 91% of studies quantified uncertainty in the predicted morbidity indicators and 23% of studies mapped it. In addition, 57% of the studies quantified uncertainty in the regression coefficients but only 7% incorporated it in the regression response variable (morbidity indicator). Fifty percent of the studies discussed uncertainty in the covariates but did not quantify it. Uncertainty was mostly defined as precision, and quantified using credible intervals by means of Bayesian approaches.

**Conclusion/Significance:**

None of the studies considered adequately all sources of uncertainties. We highlighted the need for uncertainty in the morbidity indicator and predictor variable to be incorporated into the modelling framework. Study design and spatial support require further attention and uncertainty associated with Earth observation data should be quantified. Finally, more attention should be given to mapping and interpreting uncertainty, since they are relevant to inform decisions regarding the number of people at risk as well as the geographic areas that need to be targeted with mass drug administration.

## Author Summary

In recent years spatial modelling studies of schistosome and soil-transmitted helminth infections have become commonplace; however there is no standard framework for uncertainty evaluation and reporting. In this study we aim to identify faults in existing studies and propose a framework for evaluation and reporting. We conducted a systematic review of the literature to identify the gaps in knowledge in relation to how uncertainty is dealt with in existing studies addressing the spatial modelling of helminth infections. It was found that none of the studies considered adequately all sources of uncertainty. Uncertainty in the response variables and covariates should be incorporated into the modelling framework. More attention should be given to mapping and interpreting uncertainty, and to quantify the different sources of uncertainty present in the observed covariates (environmental variables), measured response variable (morbidity indicators), used model and uncertainty representation and interpretation of the predicted morbidity indicators.

## Introduction

Helminth infections from as soil-transmitted helminths (STHs) and schistosomes are among the most prevalent neglected tropical diseases (NTDs) affecting human populations living in countries where clean water, sanitation, and hygiene (WASH) are limited. STHs and schistosomes, affect more than 1.7 billion and 252 million [1,2] people worldwide respectively. The majority of these infections are concentrated in sub-Saharan [3,4] and North Africa, Asia, and central and Andean regions of Latin America [1]. STH and schistosome infections influence directly the nutrition status, educational development, individual productivity, physical and mental development in human populations [5]. The World Health Organization (WHO), the World Bank and other agencies defined control and elimination targets in the poorest populations [6]. Although the global burden of NTDs declined by 27% from 1990 to 2010 in upper-middle income countries [6], low and lower middle income countries still need attention. Besides, according to the Global Burden of Disease Study 2010 [1], STHs due to intestinal nematode infections, and schistosomiasis, caused the largest number of cases reported in 2010. In order to improve population health and accomplish WHO targets, the 2012 London declaration for Neglected Tropical Diseases and the 2013 World Health Assembly resolution highlighted the importance of mass drug administration (MDA) with benzimidazoles [7,8] to communities at risk.

To identify communities at risk, indirect indicators of morbidity such as prevalence of infection and intensity of infection can be measured via surveying at-risk populations [9]. Communities at risk can then be categorized into disease prevalence classes (e.g. low, moderate, high) based on WHO guidelines [10]. In the absence of empirical data on infection at unsampled communities, one way to identify communities at risk is to study the role of the environment (physical and biological) to characterize potential habitats of parasites and intermediate hosts, as well as to understand the ecology and epidemiology of infections. Statistical modelling of the spatial distribution of helminth infections provides empirical relationships between infections and risk factors, which can then be used to predict the level of infection prevalence at unsampled locations [9,11–13]. In the statistical model, prevalence or another morbidity indicator, is treated as the response variable.

Although statistical modelling of helminth infections is useful to effectively and efficiently manage surveillance, control and prevention of the infection [14], the mapped outputs should be interpreted with care because these can be weakened by several sources of uncertain information [15]. Sources of uncertainty that need to be accounted for in the modelling process include differences in variable selection criteria, statistical methods used, selected spatial and temporal scales of analysis [16], sampling design, sensitivity and specificity of diagnostic techniques as well as the quality of the spatial data used.

Uncertainty has been the subject of extensive discussion in Geographic Information Science (GIScience) [17–32] and related subjects [33–43]. Uncertainty may relate to (1) a state of mind and our perception of the world or (2) statements about the world or observations on natural phenomena [17,18,22,32] and is relevant in terms of specifications and representations, measurement and the transformations, processing and modelling performed on raw data to turn them into usable information [17,22]. In order to address uncertainty, a more formal approach is required [17,18]. Here we conceptualize uncertainty as *imperfection*, which is further categorized as *inaccuracy* or *imprecision*.

Imprecision may arise because the phenomenon is vague (i.e., the phenomenon is not clearly defined), ambiguous (i.e., different definitions can be applied to the phenomenon) [23,32] or due to the granularity of the observation [17]. In the spatial setting granularity relates to the resolution or spatial support (area or volume) of the observation and affects our ability to discern objects [17,44]. Imprecision may also arise due to natural variability, measurement error and model variability and may be described statistically, for example by the variance or standard deviation [32,45,46]. In this context, model variability may arise due to uncertain data, stochastic processes within the model or variability between competing models. The reader may be familiar with the narrow statistical definition of precision as the inverse of the variance [47], whereas the imprecision that is applied here encompasses a wider set of concepts [17,18]. Put another way, in this conceptualization, variance is not the only measure of precision.

Accuracy is a measure of closeness between the observed phenomenon and reference observations, considered representative of the reality [17,45,48]. Accuracy assessment is often referred to as validation [20,49]. Common measures of accuracy include the root mean square error (RMSE) for continuous data [45,48], the overall accuracy (OA) for categorical data [27,28,50] and the area under the receiver operator characteristic curve (AUC) for binary data [45]. Bias relates to accuracy and refers to systematic differences between the observations and reference data.

Accounting for uncertainty in disease mapping is important for the assessment of the applicability and validity of the predicted morbidity indicators [15]. Furthermore, it will allow a complete risk assessment and the identification of potential sources of bias [51]. Ignoring uncertainty can lead to incorrect predictions, thus wrong estimates of disease burden, which can result in misleading public health advocacy and decisions regarding disease control. Consideration of information about uncertainty is critical for control programs, health care workers, populations at risk, and other involved users who attempt to reduce prevalence and incidence of helminth infections across the affected areas [51,52]. For example, control programs need accurate information to decide about drug distribution strategies and the frequency of treatment of the target populations. Decision makers can use information about uncertainty to target more resources (e.g., data acquisition) or to focus investigative efforts on low or highly uncertain risk areas [53,54].

This paper is a systematic review that aims at the identification of the gaps in knowledge of the different components of uncertainty associated with mapping and modelling helminth infections. It also aims at providing a basis for a complete uncertainty communication, by evaluating the impact of uncertainty on the predicted morbidity indicators. This paper starts by investigating how uncertainty is informative for decision makers, public health scientists and the affected community. It then identifies main sources of uncertainty in helminth infection mapping studies, and how uncertainties have been defined and quantified. Regarding the sources of uncertainty, their definition and quantification, the focus will be put on sources relating to Earth Observation. The significance of this paper is that it contributes to inform control programs and health workers about the importance of uncertainty in mapping and modeling helminth infections, by putting special attention on relevant sources of uncertainty, and analyzing their real influence on the predicted morbidity indicator values used to guide mass drug administration strategies and their cost effectiveness.

## Methods

### Search strategy

An online search was performed using two search engines, the Web of Knowledge (Core collection and MEDLINE) and PubMed. Only articles published in English were considered. The date range was 1 January 1980 to 24 October 2016. The search strategy aimed at the identification of primary research studies that have looked into establishing the geographical limits of STH and schistosomiasis present only in humans; therefore the search strategy combined variations of three terms: spatial, helminth infection, and uncertainty terms. The full list of terms used in the systematic review is shown in Table 1. Six searches were performed by combining the three terms in each search engine, using the keywords described in Table 2.

**Table 1:** Classification of search terms

Uncertainty term (UT)	Spatial term (ST)	Disease term (UT)
Uncertainty, uncertain, uncertainties.	Geographic, geographical, geography	helminth(s), helminthiasis, soil-transmitted helminths, soil-transmitted helminthiasis, neglected tropical diseases.
Vagueness, vague	Spatial, geospatial	Schistosome, Schistosoma, schistosomiasis.
Imprecision, precision, precise, imprecise	Remote sensing, remotely sensed	Hookworm(s)
Accuracy, inaccuracy, accurate, inaccurate		Trichuris trichiura
Fuzzy, fuzziness		Ascaris lumbricoides
Error(s)		
Bias		

**Table 2:** Keywords used in the literature search,* indicates wildcard

Uncertainty term		Spatial term		Disease term	
1	TS = uncertain*	3	TS = geogra* OR TS = spatial OR TS = geo$spatial OR TS = "remote* sens*"	4	TI = schistosom*
2	TS = vague* OR TS = *precision OR TS = *precise OR TS = *accura* OR TS = fuzz* OR TS = error* OR TS = bias			5	TI = hookworm* OR TI = "trichuris trichiura" OR TI = "ascaris lumbricoides"
				6	TI = helminth* OR TI = "soil$transmitted helminth*" OR TS = “neglected tropical disease*”

**Fig 1:**
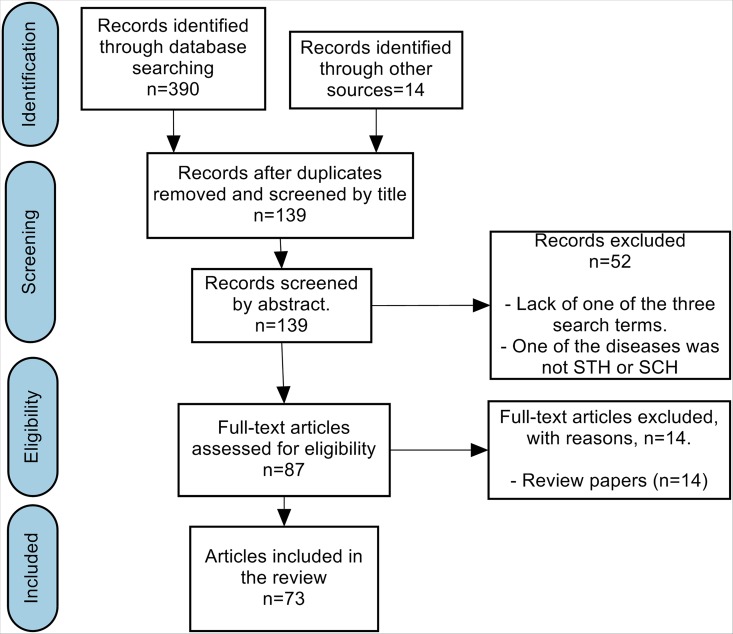
PRISMA flow diagram.

After removing duplicates, the abstracts of 139 papers were read. Papers written in languages other than English (11 papers) were automatically excluded. Review papers (14 papers) were also excluded. Further criteria were then applied to select the final papers to read, but also to make the reading process more efficient. The inclusion criteria considered were (i) the presence of the three spatial, uncertainty and helminth infection search terms in the abstracts and (ii) also articles related to only STH and schistosomiasis helminth infections. The papers were classified into schistosomiasis and soil transmitted helminth studies. The selection of the papers, data acquisition and analysis was undertaken by the first author. The PRISMA flow diagram is given in Fig 1.

### Data collection process

Data collection from each paper focused on addressing three main research questions. (1) How is uncertainty informative for decision making in the public health context? (2) What are the different uncertainty sources reported in the reviewed studies? (3) How were uncertainty and its sources defined and quantified in the studies? Papers addressing these questions were enumerated.

**Fig 2:**
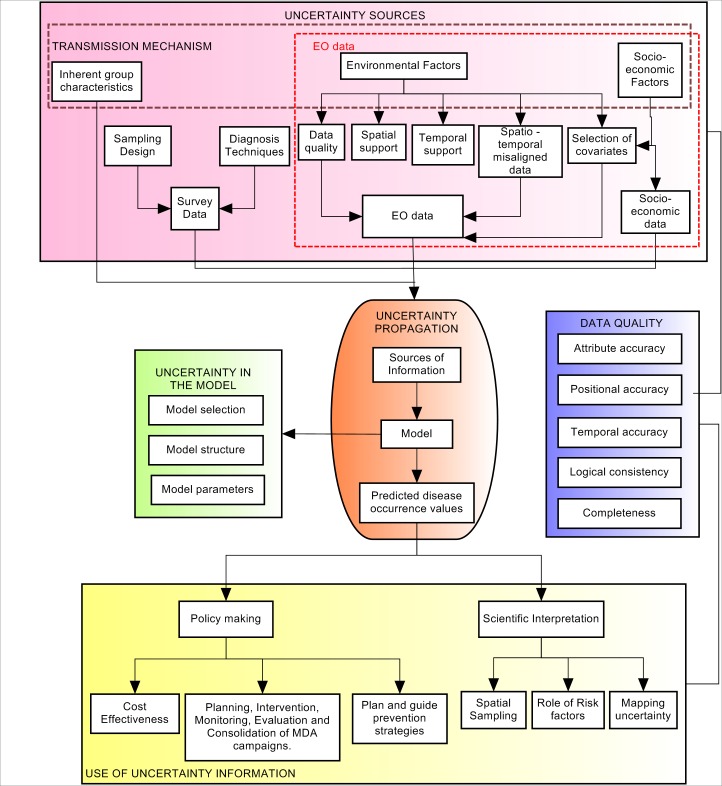
Uncertainty propagation through the process chain of mapping and modelling helminth infections. Pink box: uncertainty from information data sources. Orange box: uncertainty from the predictive model. Yellow box: uncertainty in the predictions.

Fig 2 illustrates the relevant three uncertainty stages that drive the final mapping and modelling of STH and schistosomiasis infections. The first stage (pink box) describes the origin of uncertainty coming from data sources, including uncertainties in the response variable and covariates. The second stage (orange box) shows how uncertainty from the pink box propagates through the predictive model (green box). The green box incorporates uncertainties derived from the selection of the predictive model, considering that there could be different ways to model the same helminth infection. It also includes uncertainties in model structure, which refers to all possible limitations and assumptions in the selected model, such as: the lack of understanding about the interaction between the environment, helminth infections and human populations, as well as the assumptions of stationarity and spatial isotropy [9]. Finally, the green box includes uncertainties in the methods used to estimate the model parameters. The third stage (yellow box), shows how uncertainty in the predicted morbidity indicator is addressed, firstly in policy and decision making settings and secondly in a scientific setting. This stage aims to understand how information on uncertainty is used practically and how is it defined and quantified. The blue box represents different elements of data quality that relate to the sources of information (pink box), and the predicted morbidity indicators (yellow box), which due to its wide field of study and importance was separated into a different box.

#### Uncertainty use in helminth infection mapping for morbidity control (uncertainty interpretation)

Two approaches were considered to describe the possible usage of uncertainty in helminth infections modelling. The first approach indicates that uncertainty could be used in policy making in order to support public health institutions, governments and national or international organizations involved in the control and prevention of STH and schistosome infections. Three foci of attention for policy making were considered: (1) plan and guide prevention strategies, (2) plan the intervention, monitoring, evaluation and consolidation of MDA campaigns, (3) evaluate cost-effectiveness of control programmes. The second approach proposes to use uncertainty to support scientific interpretation by looking at the influence of different information sources on the modelling process, and decide about new improvements or conclusions that need to be considered. Three foci of attention for scientific research were considered: (1) spatial sampling, (2) the role of risk factors (covariates in the statistical model), (3) the mapping of uncertainty. An overview of the different foci of attention of uncertainty information is explained in Table 3.

**Table 3:** Description of communication of uncertainty

Uncertainty informs about		Description
**Policy Making**	Planning, Intervention, Monitoring, Evaluation and Consolidation of MDA campaigns.	• *Plan* spatial targeting and the frequency of deworming campaigns to estimate required drug supplies. • Guide *interventions* towards high risk populations. • *Monitoring*: Maintain success and long term sustainability of control programs. • *Evaluation*: compare and choice more efficient strategies to control the disease. • *Consolidate* control and move towards disease elimination.
	Cost effectiveness	• Inform about the cost associated with the health benefit acquired by implementing a specific control strategy. • Ensure the resources are distributed efficiently by channel funds to high risk populations.
	Plan and guide prevention Strategies	• Plan and guide hygiene education and infrastructure programs in water sanitation and hygiene, as well as implement environmental educational health awareness programs. • Control intermediate host or parasite sources to prevent transmission to definitive hosts.
**Scientific Interpretation**	Sampling	• Define uncertain risk areas where further data collection is required. • Guarantee the safety of local citizens from future infection resurgence by determining appropriate surveys and monitoring strategies.
	Role of risk factors	• Investigate the effect of environmental risk factors on transmission of parasites. • Guide control efforts in the absence of epidemiological information.
	Mapping Uncertainties	• Spatial representation of uncertainty as a necessary resource for decision making.

#### Uncertainty sources in modelling and mapping helminth infections (uncertainty in the data)

Sources of uncertainty shown in the red box in Fig 2 were classified into four: (1) survey, (2) Earth observation, and (3) socio-economic data, (4) inherent group characteristics. Survey data encompassed uncertainties in the response variable, while Earth observation and socio-economic data were uncertainty sources coming from the covariates. Survey data contained uncertainty from the sampling design and diagnostic technique. Sampling design refers to the type of survey used, sample manipulation, sample size selection, incomplete sample coverage, logistic limitations, survey registration method, adjustment for confounding and the measured morbidity indicator. Uncertainty in the diagnostic technique arises due to the lack of sensitivity and specificity in the methods used to detect helminth parasites eggs in the stool or urine of affected individuals. Uncertainties derived from Earth observation data arise due to spatio-temporal misaligned data, incorrect selection of significant environmental and socio-economic variables, as well as selection of spatial and temporal support of analysis which do not fit the study purpose. The term *misaligned data* refers to the combination of multiple datasets that may be defined on different or non-aligned spatial units [55], whereas the support refers to size, shape and orientation of the spatial units [56]. The term *scale* can have multiple meanings in geographical information science (GIScience) [44]; here we consider scale in terms of the *support* of the data and the *extent* of the study domain [45]. Data quality refers to the evaluation in terms of fitness-for-use for a given application [11]. This evaluation addresses the completeness, logical consistency, time, attribute and positional accuracy of spatial data [57–60]. Different measurements of the same variable may even have different qualities according to the sensitivity, specificity and accuracy of the instrument or measurement technique.

Scale is a major concern in spatial epidemiology [11,45,61–63]. Different environmental and socio-economic risk factors may be relevant according to the scale of the analysis [11,64]. For a given extent the choice of support may affect the patterns identified in the data [65,66] as well as the relationship between the response variable and covariates. This is known as the modifiable areal unit problem (MAUP) in GIScience [11,44]. Different datasets may be misaligned and need to be brought to a common grid prior to analysis [66,67]. Hence it may be necessary to aggregate, disaggregate or interpolate data prior to analysis [11,68]. All of these operations may be applied in time and space and all have an associated uncertainty. Issues about the selection of significant environmental and socio-economic variables referred to: (1) the exclusion of some socio-economic and climatic factors, which due to logistics or lack of reliable information have not been included in the modelling process; (2) the uncertain choice of covariates produced by the lack of knowledge about the influence of risk factors depending on the spatial support of analysis, the spatial support of the data and other aspects of data quality. Sources of uncertainty derived from inherent group characteristics refer to the heterogeneous distribution of parasites in the population, and the influence of polyparasitism (infection due to multiple parasites also termed coinfections) on the risk of infection.

#### Uncertainty definition and quantification in helminth infections mapping

As mentioned in the introduction, uncertainty was conceptualized as imperfection and further categorized as accuracy and imprecision [17,18]. Accuracy may be evaluated by comparison with a reference dataset [17,18,27,28,45,48,50] and different quantitative measures may be used depending on the type of data. Continuous data may be evaluated using the root mean square error (RMSE) or mean absolute error (MAE), which are both measures of the average error. Bias can be evaluated using the mean error. Categorical data are typically evaluated using a confusion matrix with summary measures including the overall accuracy, user’s and producer’s accuracy and kappa statistic. Binary data may be evaluated using the area under the receiver operator curve (ROC) (AUC). Measures of accuracy are summarized in Table 4.

**Table 4:** Measures of uncertainty corresponding to different types of data.

Categories of imperfection	Types of data	Measures of uncertainty	Abbreviation
Imprecision	Continuous data	Standard deviation	SD
		Credible intervals	CrI
		Confidence Intervals	CI
	Categorical data (Vagueness)	Fuzzy sets	
		Rough sets	
Inaccuracy	Continuous data	Root mean square error	RMSE
		Mean absolute error	MAE
		Residual mean square	RME
		Mean error (bias)	ME
	Categorical data	Overall accuracy	OA
		User’s accuracy	UA
		Producer’s accuracy	PA
		Kappa statistic	K
	Binary data	Area under the receiver operator characteristic curve	AUC

Evaluation of imprecision depends on the nature of the phenomena and data being studied. Where these are well defined, imprecision may be defined statistically [21,32] and applied in both Bayesian and frequentist settings. The error variance is the usual measure here, although this is commonly expressed as the standard deviation or standard error [32] or as an interval–such as the 95% confidence interval (frequentist) or credible/credibility interval (Bayesian) [69]. Vagueness may be evaluated using fuzzy set or rough set theory [21,32]. Table 4 shows the elements and measures of uncertainty conceptualized as imperfection.

## Results

### Search strategy

**Fig 3:**
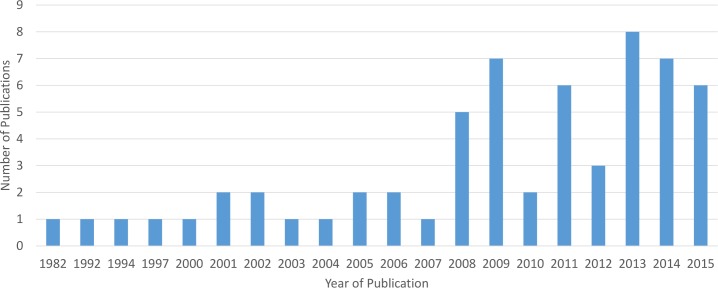
Year of publication of studies included in this review.

The total number of papers found in each search is shown in Table 5. Table 6 shows the resulting number of read and discarded papers presented per infection. In total 73 papers were selected, from which 14 were review papers. While the identified review papers were not included in this review we examined their reference lists; this yielded another 14 valuable references that had not been identified by our original search. Finally 73 primary research papers were included in our systematic review. Our results demonstrate that the annual number of publications on mapping and modelling STH and schistosome infections was constant until the year 2007 and steadily increased since then; since 2008 a total of 49 (67% of the total) papers were published (Fig 3).

**Table 5:** Results of the search performed in the Web of Knowledge and PubMed, using the search terms and the corresponding keywords given in Table 1 and Table 2 respectively.

UT	ST	DT	Results Web of Science	Results PubMed
1	3	4	24	23
2	3	4	72	65
1	3	5	0	5
2	3	5	7	18
1	3	6	19	13
2	3	6	52	90

**Table 6:** Total number of read and discarded papers presented per infection.

	Read papers	Discarded papers
**Schistosomes**	47	26
**STH**	26	26

### Data collection process

#### Uncertainty use in helminth infection mapping for morbidity control

For policy making 47 (64%) studies used uncertainty information, in planning, intervention, monitoring, evaluation and consolidation of MDA campaigns (Table 7). This was followed by 15 (21%) studies that focused on increasing cost effectiveness of these programmes. Five studies (7%) used uncertainty in disease maps to inform about prevention strategies such as to plan and guide hygiene education and infrastructure WASH programmes. For scientific interpretation, only seven studies (10%) used uncertainty to improve spatial sampling, eight studies (11%) used it to investigate the role of environmental and socio-economic risk factors on the infections, and 17 (23%) papers mapped uncertainty.

**Table 7:** Use of information on uncertainty in the public health context

Uncertainty informs about		Papers SCH	Papers STH	Total
**Policy Making**	Cost effectiveness	[66,71,77,81,98,99,103,112,130,148]	[87,88,107,108,149]	15
	Planning, intervention, monitoring, evaluation and consolidation of MDA campaigns.	[16,53,65,66,71,74–77,79–81,93,96–102,104,105,111,119,125,130–132,138,147,148,150–155]	[87,90–92,107–109,129,140,156,157]	47
	Plan and guide prevention strategies	[79,130,154]	[108,140]	5
**Scientific Interpretation**	Sampling	[71,75,80,106,119,152]	[82]	7
	Role of risk factors	[54,78,89,94,95,155]	[84,85]	8
	Mapping uncertainty	[66,70–72,74,75,77,79,80,98,105,111,119,130,131]	[108,109]	17

#### Uncertainty sources in modelling and mapping helminth infections

Table 8 shows that, from the total number of reviewed papers, sampling design was the most highlighted source of uncertainty, with a total of 42 (58%) papers acknowledging it. The second and third most highlighted sources of uncertainty were diagnostic techniques, with a total of 29 (40%) papers acknowledging it, and selection of significant environmental and socio-economic variables, acknowledged by 22 (30%) papers. The last highlighted uncertainty source was related to spatial support, with 19 (26%) papers acknowledging it. The least highlighted uncertainty sources were: inherent group characteristics, use of data with insufficient quality, temporal support, and spatio-temporal misalignment, with 15 (20%), 15, 7 (10%) and 5 (7%) papers acknowledging them respectively. From the category sampling design, the most highlighted sources of uncertainty were: incomplete sample coverage and sample size, with respectively 16 (37%) and 22 (51%) papers acknowledging them respectively (Table 9). Heterogeneity and polyparasitism were acknowledged by nine (12%) and six papers (8%) respectively

**Table 8:** Uncertainty sources in modelling and mapping helminth infections

Uncertainty sources			Papers using different measures of uncertainty	Papers highlighting the importance of uncertainty sources		Total
				Papers SCH	Papers STH	
**Input data**	**Survey Data**	Sampling design	ROC (AUC) [71]	[66,71–74,76,78–81,91,93,96,97,99–101,103–105,111,125,130–133,138,147]	[86–88,90,92,107,109,110,129,140,156,158–160]	42
			Credible intervals [101]			
		Diagnostic Techniques	Credible intervals [76,87]	[65,66,76,78,79,81,95–97,101,104–106,111,112,130–132,138,148,154,155]	[86,87,107,108,140,149,157]	29
	**EO data**	Spatial support		[71,76,77,81,95,97,103,106,111,130,131,147,154]	[84,85,108,109,156,159]	19
		Temporal support		[73,106]	[84–86,88,109]	7
		Data quality		[16,74,77,79,89,91,93,95,99,106,119]	[88,90,129,140]	15
		Spatio-temporal misaligned data		[103,154]	[119,129,140]	5
		Selection of significant environmental and socio-economic risk factors	*Credible Intervals*:	[71,76,79,81,94,101,104,111,125,130,131,133,147,150,151,154]	[86,87,107,108,140,156]	22
	**Socio-economic data**					
			SCH: [53,54,65,66,71,73,76,89,93–106,111,112,119,130,147,148,155]			
			STH: [84,85,87,88,90,107–110,140,156,157]			
			*Confidence Intervals*:			
			SCH: [73,106,138]			
			STH: [84–86,159]			
**Inherent group characteristics**	Heterogeneity		ROC (AUC) [99]	[66,76,94,99,104,148]	[107,140,160]	9
	Polyparasitism			[66,111,112,148]	[110,129]	6

**Table 9:** Categories of sources of uncertainty and papers included in this review grouped into categories

Categories	Uncertainty sources	Papers focusing in schistosomiasis	Papers focusing on STH	TOTAL
**Sampling Design**	Type of survey	[97,100,101,125,160]	[156]	6
	Samples manipulation	[138]		1
	Sample size	[66,72–74,80,100,103–105,111,125,130–132]	[86,87,107,109,110,140,156,158]	22
	Sample coverage	[76,80,93,99,105,111,130,147]	[87,88,90,92,107,129,140,159]	16
	Logistics	[78,81,99,131,133]	[86,92]	6
	Survey registration method	[71,91,103]		3
	Adjust for confounders	[101]		1
	Selection of the measure of risk	[125]	[140,160]	3
**Diagnostic Techniques**	Sensitivity and specificity of diagnostic methods	[65,66,76,78,79,81,95–97,101,104–106,111,112,130–132,138,148,154,155]	[86,87,107,108,140,149,157]	29
**Spatial support**	Spatial aggregation and disaggregation	[71,76,77,81,95,97,103,106,111,130,131,147,154]	[84,85,108,109,156,159]	19
**Temporal support**	Temporal aggregation and disaggregation	[73,106]	[84–86,88,109]	7
**Data quality**	Position accuracy, logical consistency, time accuracy, completeness, attribute accuracy (pre-processing)	[16,74,77,79,89,91,93,95,99,106,119]	[88,90,129,140]	15
**Spatio-temporal misaligned EO data**	Spatial and temporal misaligned EO data.	[103,154]	[119,129,140]	5
**Selection of environmental and socio-economic variables**	*Environmental*: Distance to water bodies, land surface temperature, soil moisture, vegetation cover, Rainfall.	[71,76,79,81,94,101,104,111,125,130,131,133,147,150,151,154]	[86,87,107,108,140,156]	22
	*Socio-Economic*: poverty, clean water, sanitation and hygiene, urbanization, land use.			
**Inherent group characteristics**	Heterogeneity	[66,76,94,99,104,148]	[107,140,160]	9
	Polyparasitism	[66,111,112,148]	[110,129]	6

Regarding uncertainty relating to the model, model structure was the most highlighted source of uncertainty, with 19 (26%) papers acknowledging it, followed by, uncertainty in model selection and uncertainty in model parameters with 3 (4%) papers each (Table 10).

**Table 10:** Model sources of uncertainty

Model uncertainty sources	Papers SCH	Papers STH	Total
Model parameters	[16,78,99]		3
Model selection	[119]	[140,160]	3
Model structure	[53,66,73,75–77,81,99–101,104,106,119,130,147,148]	[85,107,108,140]	20

#### Uncertainty definition and quantification in helminth infections mapping

Four ways to define uncertainty were found: *accuracy*, *imprecision*, *bias* and *vagueness*. Sixty-one (83%) papers expressed uncertainty in the modelled results using measures of imprecision and credible intervals were the most frequently used measure of imprecision (Table 11). Thirty-nine (53%) papers defined uncertainty by means of accuracy, using mostly the area under the curve of the receiver operating characteristic and the percentage of correctly predicted morbidity indicators. Bias and vagueness were the least used measure of uncertainty with only five (7%) and one (1%) papers quantifying uncertainty in their results by means of mean error and fuzzy sets respectively.

**Table 11:** Uncertainty definition and quantification

Uncertainty definition	Uncertainty quantification	Model + parameters		Total	Parameters	
		Papers SCH	Papers STH		Papers SCH	Papers STH
Accuracy	Residual mean square.	[77]		1		
	Mean absolute error.	[66,97,101,150]	[65,108–110]	8		
	Percentage of locations that were predicted within a 95% confidence/credible interval.	[66,89,98,101,105,112,130,148,161]	[107,109,110]	12		
	Receiving operating characteristics (AUC).	[71,76,81,93,98–100,111,119,125,147,162]	[87,90,108,140,157]	18	[71,99]	
	Point-wise standard error.	[80]		1		
	Log likelihood ratio.	[151]		1		
	Root mean square error.	[70,72,162]		3		
	Kappa statistic.	[74]	[82]	2		
Precision	Bayesian approaches (Credible Intervals).	[53,54,65,66,71,73,76,89,93–106,111,112,119,130,147,148,155]	[84,85,87,88,90,107–110,140,156,157]	42	[53,54,65,66,71,73,76,89,93–106,111,112,119,130,147,148,155]	[84,85,87,88,90,107–110,140,156,157]
	Standard deviation.	[70,75,131,153]		4		
	Standard deviational ellipse.	[79]		1		
	Frequentist approaches (Confidence intervals, R squared).	[16,53,54,66,70–81,89,91,93,95,96,106,110,130,138,154,161]	[82,84–88,90,92,94,159]	38	[53,54,66,70–81,89,91,93,95,96,106,110,130,138]	[82,84–88,90,92,94,159]
	Ranking statistic based on maximum likelihood.	[16]		1		
Bias	Residual, mean error	[65,66,70,103]	[108]	5		
Vagueness	Fuzzy theory	[163]		1		

A total of 57 (78%) studies evaluated regression coefficient parameters by means of precision, and quantified them using Bayesian approaches (57%), and frequentist approaches (52%). This overlap arose because several authors first used frequentist non-spatial approaches to identify the significant covariates [54,60,65,66,70–96] and then applied these covariates in a Bayesian geostatistical model [2,4,95,97–112]. Two papers (3%) quantified the uncertainty arising due to questionnaires data, as well as the uncertainty arising due to combining age-groups in the predictions [71,101]. Regarding diagnostic techniques, two studies (3%) addressed diagnostic uncertainty by modelling sensitivity and specificity as random variables, specified as beta distributions, and quantified as posterior credible intervals [76,87].

## Discussion

Currently, decisions about helminth control programs and their cost-effectiveness are made under uncertainty. To assist decisions about investment and allocation of disease control resources such as mass drug administration, maps depicting the geographical limits of risk are being used as decision support tools. Modern disease mapping utilizes advanced modelling frameworks to determine the endemicity of infection. There is a concern about the validity of spatial modelling frameworks in that, if spatial uncertainty is not adequately taken into account, this could result in erroneous conclusions and decisions about the spatial distribution of these diseases [51].

### Uncertainty use in helminth infections mapping for morbidity control

Most of the studies used information on uncertainty to guide MDA campaigns and evaluate their cost effectiveness. Information on uncertainty was also used to evaluate the role of risk factors in mapping helminth infections. Nevertheless, prevention strategies, improvements in sampling design, and mapping of uncertainty have not yet been addressed [113–116]. We advise to use information on uncertainty not only to inform about MDA campaigns, but also to inform about prevention strategies such as improving sanitation and hygiene education [117] or delineating potential transmission sites [116]. Transmission control is important for its public health relevance, since potential disease transmission sites could guide direct intervention measures at the place of infection [62,116]. Likewise, mapping of uncertainty is also recommended, since it is known to be an important tool for public health decision making, especially to determine the geographical distribution of areas for which information is lacking [112]. Mapping could be used as a tool to improve the sampling strategy and modelling efforts. Maps of uncertainty could also support communication of uncertainty to the affected communities. A complete exploration and judgement of uncertainty information would enhance the assessment of the risk of getting these infections, and would allow to understand potential impacts on human health [51].

While most studies identified and discussed different sources of uncertainty, this was mainly limited to a qualitative discussion, rather than a quantitative one [118] (Table 11). For instance, 38 (52%) papers highlighted qualitatively the importance of sampling design in mapping helminth infections, but only two studies (3%) have quantified their possible effects on the accuracy of the predicted morbidity indicator. An example is given by Clements et al [119], where uncertainties in the predictions were used to identify areas requiring further data collection before programme implementation. The lack of a quantitative assessment limits the utility of the findings in both policy/decision making setting and a scientific setting [51,118,120,121]. Communication of uncertainty will never be complete without an extensive quantification of uncertainties in all possible information sources [51,120,122], where model assumptions, selection of covariates and acquisition of survey data are clearly explained, either within the publication or as supplementary information.

### Uncertainty sources in modelling and mapping helminth infections

**Fig 4:**
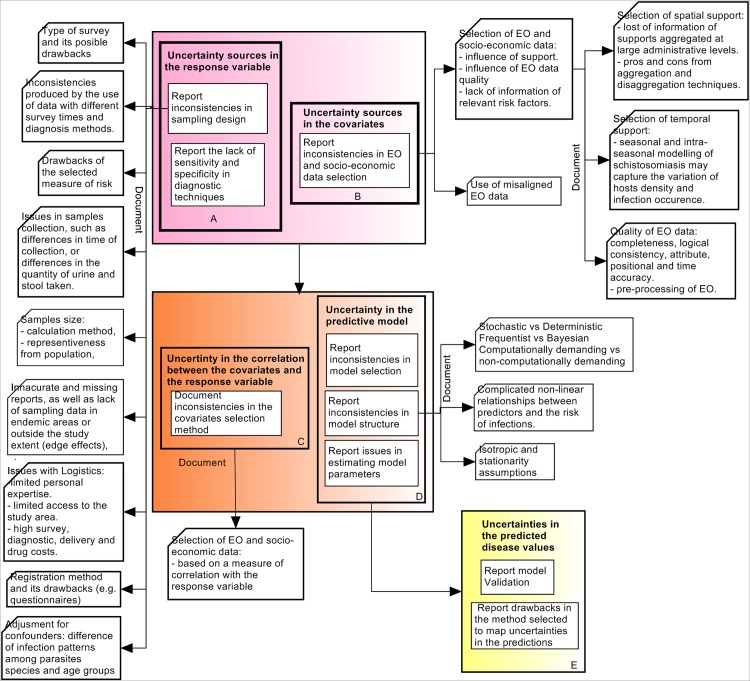
Stages of uncertainty analysis when mapping STH and schistosome helminth infections. Colour coding as for Fig 2.

Fig 4 shows the three uncertainty stages previously described in Fig 2, where these stages encompass specific uncertainty components, which need to be considered for a complete uncertainty communication. Each of these components is analyzed in the next sections.

#### Uncertainty in the response variable (morbidity indicator)

This uncertainty belongs to the first uncertainty stage (uncertainty coming from different data sources) and is described in Box A from Fig 4. This type of uncertainty exists as a function of the measurement [46] or data collection. Uncertainty in the response variable depends on the survey data quality, generated based on the sampling design, and the used diagnostic approach (Fig 2). A total of 68% of the papers mentioned the importance of sampling design as the main source of uncertainty, supporting the idea that significantly biased results may be produced due to an inappropriate sampling design [123]. When mapping helminth infections, it is suggested to document the sample size calculation method, together with the analysis of a certain target group selection. Other sources of uncertainty in sampling design are related to the type of survey, type of morbidity indicator and the use of misaligned survey data. For instance, Chammartin et al. [97] argued that cross sectional studies might not capture well the focal pattern of schistosomiasis, since their information is based on an specific point in time. Likewise, prevalence as the most frequently used morbidity indicator, underestimates morbidity values [76,124–128] and was considered a biased and poor indicator of risk [123,125]. Also, combining data from different sources of information, with different survey times and diagnosis methods may result in inaccurate estimates [66,71,100,101,129]. This is why it is suggested to document all possible drawbacks in the selected type of survey and measure of risk, and document all problems when using misaligned survey data.

Data collection also influenced the results when there was a lack of spatial and laboratory sampled data in areas where the presence of infection was suspected to be high [66,72–74,80,100,103–105,111,125,130–132]. This could be due to inaccurate and missing reports [131], lack of people’s participation [132] and limited access to geographical areas [81]. All these potential causes should be reported as well as issues regarding high costs of the survey, diagnosis, delivery of drugs, type of registration resource and limited training and expertise of field personnel, which might also influence the quality of the results [78,81,99,131,133–135]. For instance, the use of questionnaires might underestimate prevalence data, since their discriminatory performance differs among regions, and these are not always completely returned by surveyed people [71,103,136,137]. Finally, issues related to diagnostic technique, sample manipulation [135,138], and lack of stratification due to confounders [101,126,139] are also important to be considered and should also be reported and analyzed.

#### Uncertainty in the covariates (EO data)

This uncertainty is also part of the first uncertainty stage and is represented in Box B of Fig 4. Main sources of uncertainty in the covariates were related to the selection of significant environmental and socio-economic risk factors, the type of environmental data, and also to the selection of the spatial support of analysis. The importance of including risk factors such as sewage system, water supply and other climatic, demographic and socio-economic variables were the most highlighted issues (Table 8). Soares Magalhães et al [140] found that including WASH indicators as random variables in the model contributed to improved definition of the areas to target for integrated helminth control and improvement of WASH risk factors. The selection of EO data depends on the selected spatial support, defined based on the research objective and analysis method used [141,142], but also on the quality of EO data itself. In addition Walz et al. [4] argued that the relevance of environmental variables are expected to vary between different landscapes and ecological regions, having an impact on the predicted morbidity indicators. Likewise, socio-economic and ecological processes that govern schistosomiasis transmission operate and vary across different scales of observation [143,144]. Since statistical correlation can vary according to the extent of the studied area and the scale of aggregation [116,145], quantitative methods to select the optimal support of analysis, such as aggregation and disaggregation process should be documented. Clear guidance on the selection of the optimal support of EO data does not exist [11], and this remains an open topic of research. Nevertheless the choices made as well as an applied aggregation or disaggregation should be documented. Although few studies highlighted the relevance of data quality, temporal support and extent, and spatio-temporal misaligned data (Table 9), these sources of uncertainty cannot be ignored. Data quality elements (i.e completeness, logical consistency, temporal accuracy, spatial accuracy, and attribute accuracy [58]) relate to the identification of uncertainty sources, and have been shown to influence the predicted disease risk [11]. EO quality elements should also be addressed and analyzed, as well as possible inconsistencies in their pre-processing. Attention should also be put to the selection of the temporal support of analysis [146], which need to be defined depending on the study objective and the host and vectors epidemiology and ecology. Finally, both temporal and spatial supports need to be adjusted into a common temporal and spatial grid since different spatial and temporal supports, could lead to erroneous conclusions in the predictions [56].

According to our analysis, although uncertainty in the covariates has been highlighted by most studies, almost none of them have quantified their impact on the disease risk predictions, and just a few have incorporated uncertainty in the response variable. Uncertainty quantification and documentation is suggested in order to completely inform about uncertainty and help decision makers and public health scientists to undertake independent uncertainty assessments [121] and better communicate uncertainty [51,120].

#### Uncertainty in the EO data selection, predictive model and predicted disease values

Spatial prediction of parasitic disease risk patterns are explained by the statistical relationships between environmental and socio-economic covariates, individuals, and observed risk of infection [9]. Setting initial candidate environmental and socio-economic covariates and their inclusion in the predictive model is one of the first steps for geostatistical modelling of helminth infections. Thus the methods used for this selection should be explained and documented explicitly such that the statistical method itself and the measure used for covariates inclusion are clearly interpreted in the mapping process (Box C from Fig 4). The selection of the predictive model, its possible limitations (when estimating model parameters, predicting morbidity indicators, or handling non-linear relations between response variables and covariates) and assumptions made, should also be reported and justified, explaining step by step the reasoning behind the use of the specific model (Box D from Fig 4). Boxes C and D in Fig 4 relate to the green box (uncertainty in the predictive model) in Fig 2, whereas Box E relates to the model output (yellow Box from Fig 2).

The mean predicted values are often aggregated to different administrative supports, without considering the uncertainty in the predictions [147]. This could lead to a biased estimate of treatment needs [144,147]. Uncertainty can and should be incorporated into the aggregation process, yielding measures of precision (e.g., credible intervals) in the aggregated predictions. Where feasible, we advise validation of the predicted aggregated morbidity indicators (Box E in Fig 4) against empirical observations [147]. This will facilitate a more appropriate spatial target of intervention and prevention strategies.

### Conclusions

**Fig 5:**
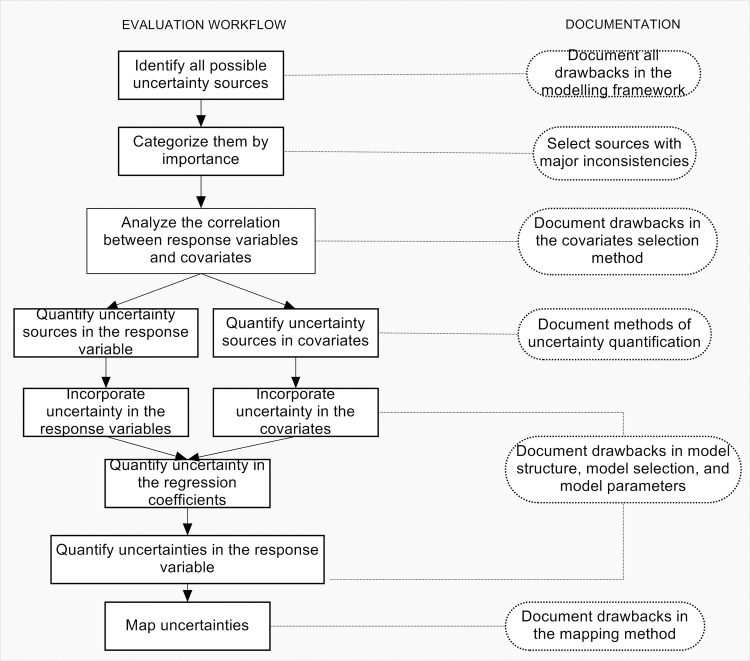
Framework for the evaluation and utilization of uncertainty in mapping soil transmitted helminth infections and schistosomiasis

Acknowledging and incorporating uncertainty in mapping and modelling helminth infections is a step-by-step process, which should be considered formally when developing geographical models of helminth infection. Geographical models aim at informing, not only about MDA campaigns and their cost-effectiveness, but also prevention strategies, where it is necessary to define transmission areas and plan and guide hygiene education and infrastructure programs in water sanitation and hygiene. A quantitative and qualitative analysis of uncertainty is necessary for a complete assessment of risk, to understand potential impacts on human health, and to allow a complete uncertainty communication to public health managers. Five components of uncertainty analysis were recognized: (1) uncertainty in the response variable, (2) uncertainty in the covariates, (3) uncertainty in the relationship between them, (4) uncertainty in the predictive model, and (5) the propagated uncertainty on the results. Our conclusions are shown diagrammatically in Fig 5, which aims at providing a framework for a full uncertainty evaluation when undertaking spatial modeling of helminth infections for policy formulation. Uncertainty analysis should start by identifying possible sources of uncertainty in the studies and categorize them such that at least the most important ones can be incorporated into the predictive model. Sampling design and EO data have been acknowledged as the major sources of uncertainty and should be given primary attention in the modelling process. In particular, sampling design, diagnosis, selection of significant risk factors, and selection of an adequate spatial support of analysis. Next, uncertainties in the response variable and covariates should be quantified and incorporated into the model. Methods used to define the relationship between covariates and response variables should also be documented, as well as the selection of the predictive model and its limitations. Finally, uncertainties in the parameters and response variables should be quantified, and uncertainty mapping should be performed as a valuable element for uncertainty communication and policy formulation.

## Supporting Information

S1 Table Prisma 2009 checklist (DOC)

S2 Table Prisma for Abstracts checklist (DOCX)

S1 Text List of papers that fulfilled the inclusion criteria and were included in the review (DOCX)

S2 Text List of papers that fulfilled the inclusion criteria but were excluded from the review for being review papers. (DOCX)
