# Supplementary material for: Mapping Soil Transmitted Helminths and Schistosomiasis under Uncertainty: A Systematic Review and Critical Appraisal of Evidence
Source: PLoS Negl Trop Dis. 2016 Dec 22;10(12):e0005208. doi: 10.1371/journal.pntd.0005208 (PMC5179027; doi:10.1371/journal.pntd.0005208)
Supplement: S2 Table — (DOCX) [file pntd.0005208.s002.docx]

**S2 Table: The PRISMA for Abstracts Checklist**

Supporting Information for

Araujo Navas AL, Hamm NAS, Soares Magalhães RJ, Stein A. Mapping Soil Transmitted Helminths Under Uncertainty: A systematic Review and Critical Appraisal of Evidence. PLoS Negl Trop Dis. Doi: 10.1371/journal.pntd.0005208

| **TITLE** | **CHECKLIST ITEM** | REPORTED ON PAGE # OR PARAGRAPH # |
| --- | --- | --- |
| 1. Title: | Identify the report as a systematic review, meta-analysis, or both. | Page 1 |
| **BACKGROUND** |  |  |
| 2. Objectives: | The research question including components such as participants, interventions, comparators, and outcomes. | Paragraph 1 |
| **METHODS** |  |  |
| 3. Eligibility criteria: | Study and report characteristics used as criteria for inclusion. | Paragraph 2: lines 1-3 |
| 4. Information sources: | Key databases searched and search dates. | Paragraph 2: lines 1-3 |
| 5. Risk of bias: | Methods of assessing risk of bias. | NA |
| **RESULTS** |  |  |
| 6. Included studies: | Number and type of included studies and participants and relevant characteristics of studies. | Paragraph 2: lines 3-4 |
| 7. Synthesis of results: | Results for main outcomes (benefits and harms), preferably indicating the number of studies and participants for each. If meta-analysis was done, include summary measures and confidence intervals. | Paragraph 2: lines 3-4 |
| 8. Description of the effect: | Direction of the effect (i.e. which group is favoured) and size of the effect in terms meaningful to clinicians and patients. | Paragraph 2: lines 4-10 |
| **DISCUSSION** |  |  |
| 9. Strengths and Limitations of evidence: | Brief summary of strengths and limitations of evidence (e.g. inconsistency, imprecision, indirectness, or risk of bias, other supporting or conflicting evidence) | Paragraph 3 |
| 10. Interpretation: | General interpretation of the results and important implications | Paragraph 3 |
| **OTHER** |  |  |
| 11. Funding: | Primary source of funding for the review. | Statement provided at submission |
| 12. Registration: | Registration number and registry name. | NA |
