# Supplementary material for: Mapping Soil Transmitted Helminths and Schistosomiasis under Uncertainty: A Systematic Review and Critical Appraisal of Evidence
Source: PLoS Negl Trop Dis. 2016 Dec 22;10(12):e0005208. doi: 10.1371/journal.pntd.0005208 (PMC5179027; doi:10.1371/journal.pntd.0005208)
Supplement: S1 Text — (DOCX) [file pntd.0005208.s003.docx]

**S1 Text: List of papers that fulfilled the inclusion criteria and were included in the review.**

Supporting Information for

Araujo Navas AL, Hamm NAS, Soares Magalhães RJ, Stein A. Mapping Soil Transmitted Helminths Under Uncertainty: A systematic Review and Critical Appraisal of Evidence. PLoS Negl Trop Dis. Doi: 10.1371/journal.pntd.0005208

**[1-73]**

1. Abdel-Rahman MS, El-Bahy MM, El-Bahy NM, Malone JB (1997) Development and Validation of a Satellites Based Geographic Information System (GIS) Model for Epidemiology of Schistosoma Risk Assessment on Snail Level in Kafr El-Sheikh Governorate. J Egypt Soc Parasitol 27: 299-316. doi:

2. Abdel-Rahman MS, El-Bahy MM, Malone JB, Thompson RA, El-Bahy NM (2001) Geographic information systems as a tool for control program management for schistosomiasis in Egypt. Acta Trop 79: 49-57. doi: 10.1016/s0001-706x(01)00102-4

3. Beck-Woerner C, Raso G, Vounatsou P, N’Goran EK, Rigo G, Parlow E, Utzinger J (2007) Bayesian Spatial Risk Prediction of Schistosoma mansoni Infection in Western Côte d’Ivoire Using a Remotely-Sensed Digital Elevation Model. Am J Trop Med Hyg 76: 956-963. doi:

4. Bisht D, Verma AK, Bharadwaj HHD (2011) Intestinal parasitic infestation among children in a semi-urban Indian population. Trop Parasitol 1: 104-107. doi: 10.4103/2229-5070.86946

5. Booth M, Bundy DAP (1992) Comparative prevalences of Ascaris lumbricoides, Trichuris trichiura and hookworm infections and the prospects for combined control. Parasitology 105: 151-157. doi: 10.1017/S003118200007380

6. Brooker S, Clements AC (2009) Spatial heterogeneity of parasite co-infection: Determinants and geostatistical prediction at regional scales. Int J Parasitol 39: 591-597. doi: 10.1016/j.ijpara.2008.10.014

7. Brooker S, Donnelly CA, Guyatt HL (2000) Estimating the number of helminthic infections in the Republic of Cameroon from data on infection prevalence in schoolchildren. Bull WHO 78: 1456-1465. doi:

8. Brooker S, Hay SI, Issae W, Hall A, Kihamia CM, Lwambo NJ, Wint W, Rogers DJ, Bundy DA (2001) Predicting the distribution of urinary schistosomiasis in Tanzania using satellite sensor data. Trop Med Int Health 6: 998-1007. doi:

9. Brooker S, Singhasivanon P, Waikagul J, Supavej S, Kojima S, Takeuchi T, Luong TV, Looareesuwan S (2003) Mapping Soil-Transmitted Helminths in Southeast Asia and Implications for Parasite Control. Southeast Asian J Trop Med Public Health 34: 24-36. doi:

10. Chammartin F, Houngbedji CA, Huerlimann E, Yapi RB, Silue KD, Soro G, Kouame FN, N'Goran EK, Utzinger J, Raso G, Vounatsou P (2014) Bayesian Risk Mapping and Model-Based Estimation of Schistosoma haematobium-Schistosoma mansoni Co-distribution in Cote d'Ivoire. PLoS Negl Trop Dis 8: e3407. doi: 10.1371/journal.pntd.0003407

11. Chammartin F, Scholte RGC, Malone JB, Bavia ME, Nieto P, Utzinger J, Vounatsou P (2013) Modelling the geographical distribution of soil-transmitted helminth infections in Bolivia. Parasit Vectors 6: 1-14. doi: 10.1186/1756-3305-6-152

12. Chan M, Medley G, Jamison D, Bundy D (1994) The evaluation of potential global morbidity attributable to intestinal nematode infections. Parasitology 109: 373-387. doi: 10.1017/S0031182000078410

13. Chen Z, Zhou X-N, Yang K, Wang X-H, Yao Z-Q, Wang T-P, Yang G-J, Yang Y-J, Zhang S-Q, Wang J, Jia T-W, Wu X-H (2007) Strategy formulation for schistosomiasis japonica control in different environmental settings supported by spatial analysis: a case study from China. Geospat Health 1: 223-231. doi: 10.4081/gh.2007.270

14. Clasen T, Boisson S, Routray P, Cumming O, Jenkins M, Ensink JHJ, Bell M, Freeman MC, Peppin S, Schmidt W-P (2012) The effect of improved rural sanitation on diarrhoea and helminth infection: design of a cluster-randomized trial in Orissa, India. Emerg Themes Epidemiol 9: 1-10. doi: 10.1186/1742-7622-9-7

15. Clements AC, Moyeed R, Brooker S (2006) Bayesian geostatistical prediction of the intensity of infection with Schistosoma mansoni in East Africa. Parasitology 133: 711-719. doi: 10.1017/S0031182006001181

16. Clements ACA, Bosque-Oliva E, Sacko M, Landoure A, Dembele R, Traore M, Coulibaly G, Gabrielli AF, Fenwick A, Brooker S (2009) A Comparative Study of the Spatial Distribution of Schistosomiasis in Mali in 1984-1989 and 2004-2006. PLoS Negl Trop Dis 3: e431. doi: 10.1371/journal.pntd.0000431

17. Clements ACA, Brooker S, Nyandindi U, Fenwick A, Blair L (2008) Bayesian spatial analysis of a national urinary schistosomiasis questionnaire to assist geographic targeting of schistosomiasis control in Tanzania, East Africa. Int J Parasitol 38: 401-415. doi: 10.1016/j.ijpara.2007.08.001

18. Clements ACA, Deville MA, Ndayishimiye O, Brooker S, Fenwick A (2010) Spatial co-distribution of neglected tropical diseases in the East African Great Lakes region: revisiting the justification for integrated control. Trop Med Int Health 15: 198-207. doi: 10.1111/j.1365-3156.2009.02440.x

19. Clements ACA, Firth S, Dembele R, Garba A, Toure S, Sacko M, Landoure A, Bosque-Oliva E, Barnett AG, Brooker S, Fenwick A (2009) Use of Bayesian geostatistical prediction to estimate local variations in Schistosoma haematobium infection in western Africa. Bull WHO 87: 921-929. doi: 10.2471/blt.08.058933

20. Clements ACA, Garba A, Sacko M, Toure S, Dembele R, Landoure A, Bosque-Oliva E, Gabrielli AF, Fenwick A (2008) Mapping the Probability of Schistosomiasis and Associated Uncertainty, West Africa. Emerging Infect Dis 14: 1629-1632. doi: 10.3201/eid1410.080366

21. Clements ACA, Lwambo NJS, Blair L, Nyandindi U, Kaatano G, Kinung'hi S, Webster JP, Fenwick A, Brooker S (2006) Bayesian spatial analysis and disease mapping: tools to enhance planning and implementation of a schistosomiasis control programme in Tanzania. Trop Med Int Health 11: 490-503. doi: 10.1111/j.1365-3156.2006.01594.x

22. Dorkenoo AM, Bronzan RN, Ayena KD, Anthony G, Agbo YM, Sognikin KSE, Dogbe KS, Amza A, Sodahlon Y, Mathieu E (2012) Nationwide integrated mapping of three neglected tropical diseases in Togo: countrywide implementation of a novel approach. Trop Med Int Health 17: 896-903. doi: 10.1111/j.1365-3156.2012.03004.x

23. Duarte HdO, Droguett EL, Moura MdC, de Souza Gomes EC, Barbosa C, Barbosa V, Araujo M (2014) An Ecological Model for Quantitative Risk Assessment for Schistosomiasis: The Case of a Patchy Environment in the Coastal Tropical Area of Northeastern Brazil. Risk Anal 34: 831-846. doi: 10.1111/risa.12139

24. Ekpo UF, Huerlimann E, Schur N, Oluwole AS, Abe EM, Mafe MA, Nebe OJ, Isiyaku S, Olamiju F, Kadiri M, Poopola TOS, Braide EI, Saka Y, Mafiana CF, Kristensen TK, Utzinger J, Vounatsou P (2013) Mapping and prediction of schistosomiasis in Nigeria using compiled survey data and Bayesian geospatial modelling. Geospat Health 7: 355-366. doi: 10.4081/gh.2013.92

25. Fonseca F, Freitas C, Dutra L, Guimaraes R, Carvalho O (2014) Spatial modeling of the schistosomiasis mansoni in Minas Gerais State, Brazil using spatial regression. Acta Trop 133: 56-63. doi: 10.1016/j.actatropica.2014.01.015

26. Gao F-h, Abe EM, Li S-z, Zhang L-j, He J-c, Zhang S-q, Wang T-p, Zhou X-n, Gao J (2014) Fine scale Spatial-temporal cluster analysis for the infection risk of Schistosomiasis japonica using space-time scan statistics. Parasit Vectors 7: 1-11. doi: 10.1186/s13071-014-0578-3

27. Hodges MH, Magalhaes RJS, Paye J, Koroma JB, Sonnie M, Clements A, Zhang Y (2012) Combined Spatial Prediction of Schistosomiasis and Soil-Transmitted Helminthiasis in Sierra Leone: A Tool for Integrated Disease Control. PLoS Negl Trop Dis 6: e1694. doi: 10.1371/journal.pntd.0001694

28. Hu Y, Bergquist R, Lynn H, Gao F, Wang Q, Zhang S, Li R, Sun L, Xia C, Xiong C, Zhang Z, Jiang Q (2015) Sandwich mapping of schistosomiasis risk in Anhui Province, China. Geospat Health 10: 111-116. doi: 10.4081/gh.2015.324

29. Kabore A, Biritwum N-K, Downs PW, Magalhaes RJS, Zhang Y, Ottesen EA (2013) Predictive vs. Empiric Assessment of Schistosomiasis: Implications for Treatment Projections in Ghana. PLoS Negl Trop Dis 7: e2051. doi: 10.1371/journal.pntd.0002051

30. Karagiannis-Voules D-A, Biedermann P, Ekpo UF, Garba A, Langer E, Mathieu E, Midzi N, Mwinzi P, Polderman AM, Raso G (2015) Spatial and temporal distribution of soil-transmitted helminth infection in sub-Saharan Africa: a systematic review and geostatistical meta-analysis. Lancet Infect Dis 15: 74-84. doi: 10.1016/S1473-3099(14)71004-7

31. Krauth SJ, Coulibaly JT, Knopp S, Traore M, N'Goran EK, Utzinger J (2012) An In-Depth Analysis of a Piece of Shit: Distribution of Schistosoma mansoni and Hookworm Eggs in Human Stool. PLoS Negl Trop Dis 6: e1969. doi: 10.1371/journal.pntd.0001969

32. Liu Z, Li C, Tang L, Zhou X, Ma L, Liu C. Prediction of oncomelania hupensis (vector of schistosomiasis) distribution based on remote sensing data and fuzzy information theory; 2015. IEEE. pp. 4408-4411.

33. Malone JB, Yilma JM, McCarroll JC, Erko B, Mukaratirwa S, Zhou XY (2001) Satellite climatology and the environmental risk of Schistosoma mansoni in Ethiopia and east Africa. Acta Trop 79: 59-72. doi: 10.1016/s0001-706x(01)00103-6

34. Martins-Bede FT, Dutra LV, Freitas CC, Guimardes RJPS, Amaral RS, Drummond SC, Carvalho OS (2010) Schistosomiasis risk mapping in the state of Minas Gerais, Brazil, using a decision tree approach, remote sensing data and sociological indicators. Mem Inst Oswaldo Cruz 105: 541-548. doi: 10.1590/s0074-02762010000400033

35. Martins-Bede FT, Freitas CC, Dutra LV, Sandri SA, Fonseca FR, Drummond IN, Souza Guimaraes RJdP, Amaral RS, Carvalho OS (2009) Risk Mapping of Schistosomiasis in Minas Gerais, Brazil, Using MODIS and Socioeconomic Spatial Data. IEEE Trans Geosci Remote Sens 47: 3899-3908. doi: 10.1109/tgrs.2009.2028332

36. McCreesh N, Nikulin G, Booth M (2015) Predicting the effects of climate change on Schistosoma mansoni transmission in eastern Africa. Parasit Vectors 8: 1-9. doi: 10.1186/s13071-014-0617-0

37. Medina DC, Findley SE, Doumbia S (2008) State-Space Forecasting of Schistosoma haematobium Time-Series in Niono, Mali. PLoS Negl Trop Dis 2: e276. doi: 10.1371/journal.pntd.0000276

38. Nihei N, Komagata O, Kobayashi M, Saitoh Y, Mochizuki K-i, Nakamura S (2009) Spatial Analysis and Remote Sensing for Monitoring Systems of Oncomelania nosophora Following the Eradication of Schistosomiasis Japonica in Yamanashi Prefecture, Japan. Jpn J Infect Dis 62: 125-132. doi:

39. Pullan RL, Bethony JM, Geiger SM, Cundill B, Correa-Oliveira R, Quinnell RJ, Brooker S (2008) Human Helminth Co-Infection: Analysis of Spatial Patterns and Risk Factors in a Brazilian Community. PLoS Negl Trop Dis 2: e352. doi: 10.1371/journal.pntd.0000352

40. Pullan RL, Gething PW, Smith JL, Mwandawiro CS, Sturrock HJW, Gitonga CW, Hay SI, Brooker S (2011) Spatial Modelling of Soil-Transmitted Helminth Infections in Kenya: A Disease Control Planning Tool. PLoS Negl Trop Dis 5: e958. doi: 10.1371/journal.pntd.0000958

41. Raso G, Li Y, Zhao Z, Balen J, Williams GM, McManus DP (2009) Spatial Distribution of Human Schistosoma japonicum Infections in the Dongting Lake Region, China. PLoS One 4: e6947. doi: 10.1371/journal.pone.0006947.

42. Raso G, Matthys B, N'goran E, Tanner M, Vounatsou P, Utzinger J (2005) Spatial risk prediction and mapping of Schistosoma mansoni infections among schoolchildren living in western Côte d'Ivoire. Parasitology 131: 97-108. doi: 10.1017/S0031182005007432

43. Raso G, Vounatsou P, Gosoniu L, Tanner M, N'Goran EK, Utzinger J (2006) Risk factors and spatial patterns of hookworm infection among schoolchildren in a rural area of western Côte d'Ivoire. Int J Parasitol 36: 201-210. doi: 10.1016/j.ijpara.2005.09.003

44. Raso G, Vounatsou P, McManus DP, Utzinger J (2007) Bayesian risk maps for Schistosoma mansoni and hookworm mono-infections in a setting where both parasites co-exist. Geospat Health 2: 85-96. doi: 10.4081/gh.2007.257

45. Raso G, Vounatsou P, Singer BH, Eliézer K, Tanner M, Utzinger J (2006) An integrated approach for risk profiling and spatial prediction of Schistosoma mansoni–hookworm coinfection. Proc Natl Acad Sci USA 103: 6934-6939. doi: 10.1073/pnas.0601559103

46. Saathoff E, Olsen A, Kvalsvig JD, Appleton CC, Sharp B, Kleinschmidt I (2005) Ecological Covariates of Ascaris lumbricoides Infection in Schoolchildren from Rural KwaZulu‐Natal, South Africa. Trop Med Int Health 10: 412-422. doi: 10.1111/j.1365-3156.2005.01406.x

47. Saathoff E, Olsen A, Sharp B, Kvalsvig JD, Appleton CC, Kleinschmidt I (2005) Ecologic Covariates of Hookworm Infection and Reinfection in Rural Kwazulu-natal/South Africa: A Geographic Information System–Based Study. Am J Trop Med Hyg 72: 384-391. doi:

48. Scholte RGC, Gosoniu L, Malone JB, Chammartin F, Utzinger J, Vounatsou P (2014) Predictive risk mapping of schistosomiasis in Brazil using Bayesian geostatistical models. Acta Trop 132: 57-63. doi: 10.1016/j.actatropica.2013.12.007

49. Scholte RGC, Schur N, Bavia ME, Carvalho EM, Chammartin F, Utzinger J, Vounatsou P (2013) Spatial analysis and risk mapping of soil-transmitted helminth infections in Brazil, using Bayesian geostatistical models. Geospat Health 8: 97-110. doi: 10.4081/gh.2013.58

50. Schuele SA, Clowes P, Kroidl I, Kowuor DO, Nsojo A, Mangu C, Riess H, Geldmacher C, Laubender RP, Mhina S, Maboko L, Loescher T, Hoelscher M, Saathoff E (2014) Ascaris lumbricoides Infection and Its Relation to Environmental Factors in the Mbeya Region of Tanzania, a Cross-Sectional, Population-Based Study. PLoS One 9: e92032. doi: 10.1371/journal.pone.0092032

51. Schur N, Gosoniu L, Raso G, Utzinger J, Vounatsou P (2011) Modelling the geographical distribution of co‐infection risk from single‐disease surveys. Stat Med 30: 1761-1776. doi: 10.1002/sim.4243

52. Schur N, Huerlimann E, Garba A, Traore MS, Ndir O, Ratard RC, Tchuente L-AT, Kristensen TK, Utzinger J, Vounatsou P (2011) Geostatistical Model-Based Estimates of Schistosomiasis Prevalence among Individuals Aged <= 20 Years in West Africa. PLoS Negl Trop Dis 5: e1194. doi: 10.1371/journal.pntd.0001194

53. Schur N, Huerlimann E, Stensgaard A-S, Chimfwembe K, Mushinge G, Simoonga C, Kabatereine NB, Kristensen TK, Utzinger J, Vounatsou P (2013) Spatially explicit Schistosoma infection risk in eastern Africa using Bayesian geostatistical modelling. Acta Trop 128: 365-377. doi: 10.1016/j.actatropica.2011.10.006

54. Schur N, Utzinger J, Vounatsou P (2011) Modelling age-heterogeneous Schistosoma haematobium and S.mansoni survey data via alignment factors. Parasit Vectors 4: 1-10. doi: 10.1186/1756-3305-4-142

55. Scott D, Senker K, England EC (1982) Epidemiology of human Schistosoma-haematobium infection around Volta Lake, Ghana, 1973-75 Bull WHO 60: 89-100. doi:

56. Seto E, Liang S, Qiu D, Gu X, Spear RC (2001) A Protocol for Geographically Randomized Snail Surveys in Schistosomiasis Fieldwork Using the Global Positioning System. Am J Trop Med Hyg 64: 98-99. doi:

57. Seto E, Xu B, Liang S, Gong P, Wu WP, Davis G, Qiu DC, Gu XG, Spear R (2002) The Use of Remote Sensing for Predictive Modeling of Schistosomiasis in China. Photogramm Eng Remote Sensing 68: 167-174. doi:

58. Soares Magalhães RJ, Barnett AG, Clements ACA (2011) Geographical analysis of the role of water supply and sanitation in the risk of helminth infections of children in West Africa. Proc Natl Acad Sci U S A 108: 20084-20089. doi: 10.1073/pnas.1106784108

59. Soares Magalhães RJ, Biritwum N-K, Gyapong JO, Brooker S, Zhang Y, Blair L, Fenwick A, Clements A (2011) Mapping Helminth Co-Infection and Co-Intensity: Geostatistical Prediction in Ghana. PLoS Negl Trop Dis 5: e1200. doi: 10.1371/journal.pntd.0001200

60. Soares Magalhães RJ, Salamat MS, Leonardo L, Gray DJ, Carabin H, Halton K, McManus DP, Williams GM, Rivera P, Saniel O (2014) Geographical distribution of human Schistosoma japonicum infection in the Philippines: tools to support disease control and further elimination. Int J Parasitol 44: 977-984. doi: 10.1016/j.ijpara.2014.06.010.

61. Soares Magalhães RJ, Salamat MS, Leonardo L, Gray DJ, Carabin H, Halton K, McManus DP, Williams GM, Rivera P, Saniel O, Hernandez L, Yakob L, McGarvey ST, Clements ACA (2015) Mapping the Risk of Soil-Transmitted Helminthic Infections in the Philippines. PLoS Negl Trop Dis 9: e0003915-e0003915. doi: 10.1371/journal.pntd.00039115

62. Souza Guimaraes RJdP, Freitas CC, Dutra LV, Carvalho Scholte RG, Martins-Bede FT, Fonseca FR, Amaral RS, Drummonds SC, Felgueiras CA, Oliveira GC, Carvalho OS (2010) A geoprocessing approach for studying and controlling schistosomiasis in the state of Minas Gerais, Brazil. Mem Inst Oswaldo Cruz 105: 524-531. doi: 10.1590/s0074-02762010000400030

63. Spear RC (2012) Internal versus external determinants of Schistosoma japonicum transmission in irrigated agricultural villages. J R Soc Interface 9: 272-282. doi: 10.1098/rsif.2011.0285

64. Spear RC, Hubbard A, Liang S, Seto E (2002) Disease Transmission Models for Public Health Decision Making: Toward an Approach for Designing Intervention Strategies for Schistosomiasis japonica. Environ Health Perspect 110: 907-915. doi: 10.1007/978-1-4419-6064-1_12

65. Sturrock HJW, Picon D, Sabasio A, Oguttu D, Robinson E, Lado M, Rumunu J, Brooker S, Kolaczinski JH (2009) Integrated Mapping of Neglected Tropical Diseases: Epidemiological Findings and Control Implications for Northern Bahr-el-Ghazal State, Southern Sudan. PLoS Negl Trop Dis 3: e537. doi: 10.1371/journal.pntd.0000537

66. Sturrock HJW, Pullan RL, Kihara JH, Mwandawiro C, Brooker SJ (2013) The Use of Bivariate Spatial Modeling of Questionnaire and Parasitology Data to Predict the Distribution of Schistosoma haematobium in Coastal Kenya. PLoS Negl Trop Dis 7: e2016. doi: 10.1371/journal.pntd.0002016

67. Tarafder MR, Balolong E, Carabin H, Belisle P, Tallo V, Joseph L, Alday P, Gonzales RO, Riley S, Olveda R, McGarvey ST (2006) A cross-sectional study of the prevalence of intensity of infection with Schistosoma japonicum in 50 irrigated and rain-fed villages in Samar Province, the Philippines. BMC Public Health 6: 1-10. doi: 10.1186/1471-2458-6-61

68. Vounatsou P, Raso G, Tanner M, N'Goran EK, Utzinger J (2009) Bayesian geostatistical modelling for mapping schistosomiasis transmission. Parasitology 136: 1695-1705. doi: 10.1017/s003118200900599x

69. Wang X-H, Zhou X-N, Vounatsou P, Chen Z, Utzinger J, Yang K, Steinmann P, Wu X-H (2008) Bayesian Spatio-Temporal Modeling of Schistosoma japonicum Prevalence Data in the Absence of a Diagnostic 'Gold' Standard. PLoS Negl Trop Dis 2: e250. doi: 10.1371/journal.pntd.0000250

70. Woodhall DM, Wiegand RE, Wellman M, Matey E, Abudho B, Karanja DMS, Mwinzi PMN, Montgomery SP, Secor WE (2013) Use of Geospatial Modeling to Predict Schistosoma mansoni Prevalence in Nyanza Province, Kenya. PLoS One 8: e71635. doi: 10.1371/journal.pone.0071635

71. Yang K, Li W, Sun L-P, Huang Y-X, Zhang J-F, Wu F, Hang D-R, Steinmann P, Liang Y-S (2013) Spatio-temporal analysis to identify determinants of Oncomelania hupensis infection with Schistosoma japonicum in Jiangsu province, China. Parasit Vectors 6: 1-8. doi: 10.1186/1756-3305-6-138

72. Zhang ZJ, Carpenter TE, Lynn HS, Chen Y, Bivand R, Clark AB, Hui FM, Peng WX, Zhou YB, Zhao GM, Jiang QW (2009) Location of active transmission sites of Schistosoma japonicum in lake and marshland regions in China. Parasitology 136: 737-746. doi: 10.1017/s0031182009005885

73. Zhang ZJ, Davies TM, Gao J, Wang Z, Jiang Q-W (2013) Identification of high-risk regions for schistosomiasis in the Guichi region of China: an adaptive kernel density estimation-based approach. Parasitology 140: 868-875. doi: 10.1017/s0031182013000048
