# Supplementary material for: Mapping Soil Transmitted Helminths and Schistosomiasis under Uncertainty: A Systematic Review and Critical Appraisal of Evidence
Source: PLoS Negl Trop Dis. 2016 Dec 22;10(12):e0005208. doi: 10.1371/journal.pntd.0005208 (PMC5179027; doi:10.1371/journal.pntd.0005208)
Supplement: S2 Text — (DOCX) [file pntd.0005208.s004.docx]

**S2 Text: List of papers that fulfilled the inclusion criteria but were excluded from the review for being review papers.**

Supporting Information for

Araujo Navas AL, Hamm NAS, Soares Magalhães RJ, Stein A. Mapping Soil Transmitted Helminths Under Uncertainty: A systematic Review and Critical Appraisal of Evidence. PLoS Negl Trop Dis. Doi: 10.1371/journal.pntd.0005208

**[1-14]**

1. Bergquist R, Tanner M (2010) Controlling Schistosomiasis in Southeast Asia: A Tale of Two Countries. In: Zhou XN, Bergquist R, Olveda R, Utzinger J, editors. Advances in Parasitology, Vol 72: Important Helminth Infections in Southeast Asia: Diversity and Potential for Control and Elimination, Pt A. pp. 109-144.

2. Boatin BA, Wurapa FK, Ulrich AM (1985) The Prevalence and Distribution of Schistosomiasis in Zambia. Cent Afr J Med 31: 170-176. doi:

3. Brooker S (2007) Spatial epidemiology of human schistosomiasis in Africa: risk models, transmission dynamics and control. Trans R Soc Trop Med Hyg 101: 1-8. doi: 10.1016/j.trstmh.2006.08.004

4. Brooker S, Kabatereine NB, Gyapong JO, Stothard JR, Utzinger J (2009) Rapid mapping of schistosomiasis and other neglected tropical diseases in the context of integrated control programmes in Africa. Parasitology 136: 1707-1718. doi: 10.1017/s0031182009005940

5. Brooker S, Michael E (2000) The potential of geographical information systems and remote sensing in the epidemiology and control of human helminth infections. Adv Parasitol 47: 245-288. doi: 10.1016/S0065-308X(00)47011-9

6. Brooker S, Clements ACA, Bundy DAP (2006) Global Epidemiology, Ecology and Control of Soil-Transmitted Helminth Infections. Adv Parasitol 62: 221-261. doi: 10.1016/s0065-308x(05)62007-6

7. Bundy DA, Hall A, Medley G, Savioli L (1992) Evaluating Measures to Control Intestinal Parasitic Infections. World Health Stat Q 45: 168-179. doi:

8. Butterworth AE (1990) Studies on Human Schistosomiasis - Chemotherapy, Immunity and Morbidity. Ann Parasitol Hum Comp 65: 53-57. doi:

9. Duarte HO, Droguett EL, Moura MC, Gomes E, Barbosa C, Barbosa V (2014) Model-based risk assessment for schistosomiasis in a Brazilian context. In: Steenbergen R, VanGelder P, Miraglia S, Vrouwenvelder A, editors. Safety, Reliability and Risk Analysis: Beyond the Horizon. London: Taylor & Francis pp. 2399-2405.

10. Hamm NAS, Soares Magalhães RJ, Clements ACA (2015) Earth Observation, Spatial Data Quality, and Neglected Tropical Diseases. PLoS Negl Trop Dis 9: e0004164. doi: 10.1371/journal.pntd.0004164

11. Manyangadze T, Chimbari MJ, Gebreslasie M, Mukaratirwa S (2015) Application of geo-spatial technology in schistosomiasis modelling in Africa: a review. Geospat Health 10: 99-110. doi: 10.4081/gh.2015.326

12. Soares Magalhães RJ, Clements ACA, Patil AP, Gething PW, Brooker S (2011) The Applications of Model-Based Geostatistics in Helminth Epidemiology and Control. Adv Parasitol 74: 267-296. doi: 10.1016/b978-0-12-385897-9.00005-7

13. Utzinger J, Raso G, Brooker S, de Savigny D, Tanner M, Ornbjerg N, Singer BH, N'Goran EK (2009) Schistosomiasis and neglected tropical diseases: towards integrated and sustainable control and a word of caution. Parasitology 136: 1859-1874. doi: 10.1017/S0031182009991600

14. Walz Y, Wegmann M, Dech S, Raso G, Utzinger J (2015) Risk profiling of schistosomiasis using remote sensing: approaches, challenges and outlook. Parasit Vectors 8: 1. doi: 10.1186/s13071-015-0732-6
